# A Fabric-Based Textile Stretch Sensor for Optimized Measurement of Strain in Clothing

**DOI:** 10.3390/s20247323

**Published:** 2020-12-20

**Authors:** Yetanawork Teyeme, Benny Malengier, Tamrat Tesfaye, Lieva Van Langenhove

**Affiliations:** 1 Department of Materials, Textiles and Chemical Engineering, Faculty of Engineering and Architecture, Ghent University, B-9052 Ghent, Belgium; Benny.Malengier@UGent.be (B.M.); Lieva.VanLangenhove@UGent.be (L.V.L.); 2 Ethiopian Institute of Textile and Fashion Technology (EiTEX), Bahir Dar University, 6000 Bahir Dar, Ethiopia; tamrat.tesfaye@bdu.edu.et

**Keywords:** conductive fabric, extension/elongation, strain sensor, resistivity, sensitivity

## Abstract

Fabric stretch sensors are available as planar fabrics, but their reliability and reproducibility are low. To find a good working setup for use in an elastic sports garment, the design of such sensors must be optimized. The main purpose of this study was to develop resistive strain sensors from stretchable conductive fabric and investigating the influence of stretchability on conductivity/resistivity. The influence of using the sensor in a sweat rich environment was also determined, in order to evaluate the potential use of the sensor in sporting garments. The sensor resistivity performance was analyzed for its sensitivity, working range, and repeatability and it was determined what makes the sensitivity when elongated or stretched. The resistivity was found to decrease with elongation if no sweat is present, this can be due to molecular rearrangement and a higher degree of orientation that improves the conductivity of a material. The result from this finding also shows that for wearable applications the commercial EeonTex^TM^ conductive stretchable fabric did not show a considerable resistivity increase, nor a good sensitivity. The sensitivity of the sensor was between 0.97 and 1.28 and varies with different elongation %. This may be due to the mechanical deformation characteristics of knitted samples that lead to changes in conductivity. We advise that the testing performed in this paper is done by default on new stretch sensitive textile materials, so practical use of the material can be correctly estimated.

## 1. Introduction

Textiles and electronics are converging (e-textiles) [1], which is relevant for the development of smart materials that are capable of accomplishing a wide spectrum of functions in textiles, which now are only found in rigid and non-flexible electronic products. Currently, wearable e-textile technologies are facing exponential growth. Gradually new textiles come to the market with functionalities such as heat regulation, luminescence, touch detection [2], and sensitivity to physical characteristics [3]. Those functionalities are useful for several applications in a wide range of fields.

Recent advances in wearable devices including textile-based strain sensors have shown substantial promise for applications in health care areas as they can assist in remote health monitoring [4] and in sport (clothing that can track body movements and activity monitoring) [5]. They are used for communication, sensing and monitoring, even for position location, to enable personal applications such as protection and safety functionality, emergency response, and dedicated tasks such as controlling the vibration of muscles during athletic activities [6,7]. There are smart materials that regulate body temperature, help during rehabilitation and provide increased ergonomics. However, the full potential of wearable devices for commercial purposes still needs improvements in sensitivity, stretchability and flexibility.

The recent upsurge in using wearable personalized devices has made it progressively more important to have flexible textile-based strain sensor alternatives that can be comfortably worn and can sense broad varieties of the human physique and respond to large and small joint movements, like bending, rotation and stretching (e.g., for posture movements and breathing) [8,9,10,11,12]. Due to convenience in wearing and level of comfort while measuring human biosignals and monitoring human motion, flexible electronics like stretchable strain sensors have been developed and extensively tested [11,13,14,15,16]. Despite the increasing research into textile-based sensors, there remains a great deal of room for improvement in terms of market presence. The comfort and washability, mechanical environment, and power supply are some of the critical challenges facing smart textile. The imbalanced contributions from electronic and clothing industries result in incompletely integrated applications [16]. Furthermore, lack of esthetic appeal, design flexibility, quality standards, understanding real human and societal needs, selling performance and consumer feedback, are all challenges contributing to the above disparities [17].

A stretchable conductive fabric changes resistance when stress/strain is applied [18], so it works as a strain sensor, but it also works as a force sensing resistor if the strain the result of a force and the Young’s’ modulus of the fabric is known. Many studies have investigated theoretical and practical relationships between the electrical resistance and elongation of conductive fabrics [19,20,21] and found that resistance of a conductor was affected when the conductive material was stretched. It is well known that when a conductive material is stretched its cross-section is reduced, leading to greater resistance. However, in textiles, the higher contact pressure between filaments might lead to lower contact resistances. The relative dominance of each effect is dependent on the stitch dimension of the yarn in knitted fabrics. The different fabric directions also affect the elongation, and hence the strain, differently.

Textile-based sensors are always made of textiles and define themselves through their textile structure. There are different ways to produce electrically conductive fabrics used to manufacture textile sensors. These types of sensors can be manufactured at all fabric structure levels, i.e., yarn, fiber or coatings. Over the past decade, many techniques and materials have been used in order to realize textile sensors. Some approaches are sewn and embroidered with conductive thread/fabrics, or they can be painted or screen printed with conductive inks [22,23] or conductive polymers [24]. One method is to integrate conductive yarns in a textile structure, e.g., by weaving [25,26]. However, the integration of conductive yarns in a structure is complex. These sensors can also combine different layers of both conductive and non-conductive textiles. Other methods present microfiber sensors working as a capacitive strain sensor, while piezoelectric materials [27] are used to create fiber strain sensors that can detect bending and rotation deformations [28]. There are also stretchable conductive threads that measure variations of resistance using materials coated with conductive polymers such as PEDOT [29].

It is generally known that a variety of fibers, yarns, and fabrics are ideal candidates for use in wearable devices. With integrated superior elasticity and recovery performances elastic conductive webbing [30] or highly flexible fabrics [31] have drawn some attention. Stretch sensor properties are closely related to their fabrication and structure [11], and knitted structures perform best for this. We are hence mainly interested in knitted sensors that change their electrical resistance under strain. Therefore, these fabrics can be used as strain sensors which convert physical deformation into electrical signals. However, there are several key factors that should be taken into account, including the sensitivity, flexibility, and stretchability, during the fabrication of a strain sensor.

In this study, our objective is to present a stretchable conductive fabric sensor from a commercially available conductive stretchable fabric aimed at pressure and stretch sensor applications. The optimal design of such sensor is investigated in different types of sample length, having as criteria the ability to measure strain and electromechanical properties. Hence, we described first the sensor materials and the textile integration. Thereafter the experimental set up is given, and the influence of stretchability/elongation on resistance was investigated. We finish with the outcome and suggest where future efforts can be directed.

## 2. Materials and Methods

### 2.1. Material Used for Sensor Design Sensor Design

- *EeonTex^TM^ knitted conductive fabric (Eeonyx Corp., Pinole, CA 4564, USA)*: For the development of a resistive strain sensor a knitted conductive stretchable fabric, 0.38 mm thick, with a mass per unit area of 113.78 g/m^2^, an elongation of 40% at break and with an 85% warp recovery after stretching, is used. This fabric is available in the market as EeonTex^TM^ conductive stretchable fabric and has as composition 72% nylon and 28% spandex with a proprietary conductive formulation as a coating.
- *Silver-plated conductive thread*: A silver-plated conductive thread, MADEIRA yarn (dtex 290 ± 6 HC40), is used to sew conductive connections to measuring devices.
- *Base fabric*: 1 × 1 interlock, nylon/spandex knitted fabric with 19 wales/cm and 29 course/cm density fabric was used as a base fabric.
- *Normal sewing thread*: The sensor fabric was attached with temporary stitch on the base fabric edges using normal sewing thread.

### 2.2. Assembling of the Sensor

**Figure 1:**
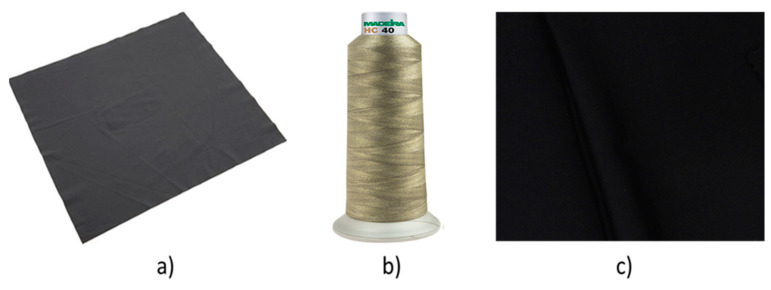
List of material used for sensor development: (**a**) conductive stretchable fabric, (**b**) silver-plated conductive thread, (**c**) nylon/spandex knitted fabric.

The sensor was designed to measure elongations in textiles. Therefore, the sensor fabric described in Section 2.1 (Figure 1a) needs to be integrated or attached to a textile. For connecting the sensor to a measurement system, the silver-coated conductive thread was used. This 100% polyamide fully silver-plated thread has linear resistance <300 Ohm/m and is suitable for robust circuitry and all applications where a very low resistance is required. In this work, polyamide fully silver-plated thread is used for optimizing contact points and circuit paths.

**Figure 2:**
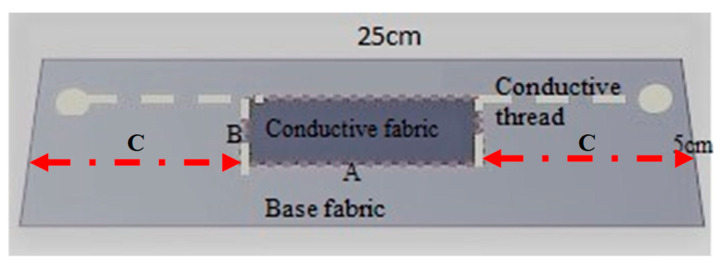
Assembled test specimen on the base fabric of 25 cm (C + A + C).

**Figure 3:**
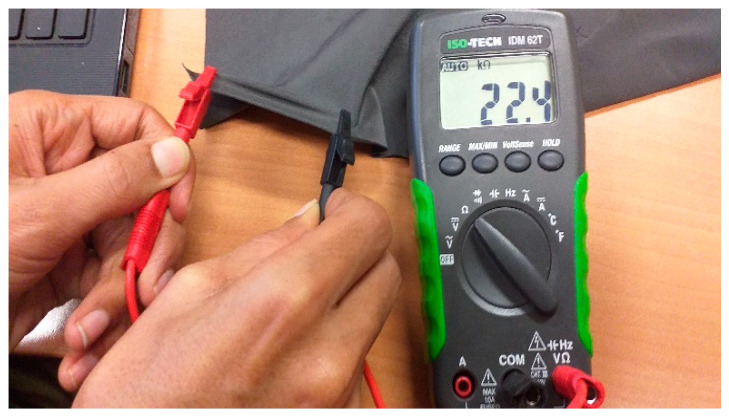
A test for identifying the warp and course direction of conductive stretchable fabric.

To keep the elasticity of the fabrics, the thread was sewn using loose long running stitch (saddle stitch). Figure 2 represents a schematic of the process of assembling of the sensor. All the necessary pieces of samples were prepared with dimensions of 25 cm × 5 cm base fabric and A × B for conductive fabric. Beforehand, the conductive fabric was tested to determine if the change of resistivity is maximum under A or B direction (Figure 3). Three replicate samples were made for the conductive fabric as per their sample size (Table 1).

The warp and course direction of conductive stretchable fabrics were identified and assigned “A” for wales and “B” for course direction and the maximum sensitivity direction was A. Samples from the conductive fabric were prepared as per the size given in Table 1. These sizes were chosen to allow measurement of human movement. Longer or wider samples would no longer be useful for this application.

**Figure 4:**
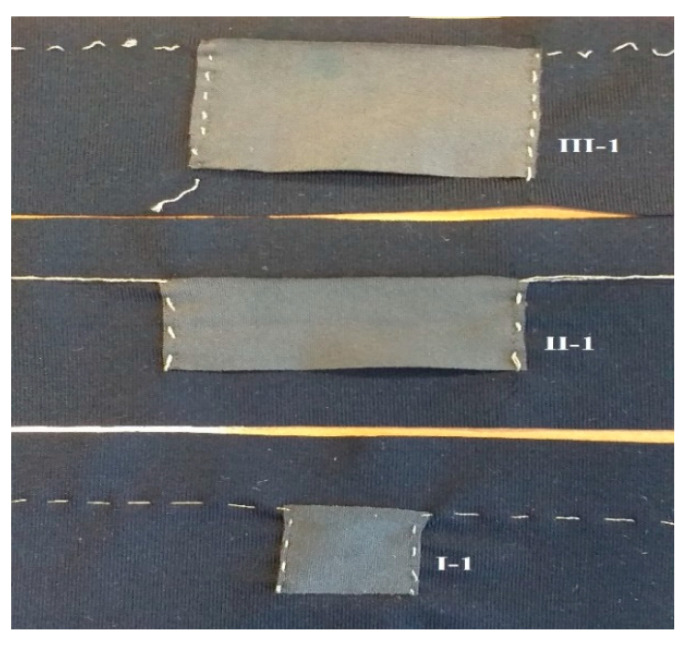
Test specimens with dimension.

A rectangle in the centre of the base fabric was drawn with the longest side in the course direction to indicate where the conductive fabric would be placed. In addition, two vertical lines (B) were placed to the left and right on the base fabric A apart. This was used later in the experiment as a location to fix the samples in the tensile test. A conductive fabric piece is placed and attached on top of the centre rectangle piece of fabric with pins under light tension in order to fix the piece and to prevent shifting when sewing (Figure 4). Thereafter, using normal sewing thread, the sensor fabric was attached with temporary stitch on the base fabric edges leaving a border of at least 3 mm, after which the pins are removed. The sensor is then connected with the conductive thread on the drawn edge of the sensor using hand stitch. This conductive thread connects to the end of the base fabric, for connection to the multimeter later. After this, the temporary stitch with normal sewing thread is removed, and the textile strain sensor is completed. Each sample was given the test number as in Table 1 (I, II, II) and the repeat number (1, 2, 3) for every realization of the specific type, e.g., I-1, I-2, etc.

### 2.3. Experimental Setup and Digital Image Analysis

To evaluate the performance of the sensors for use in measuring strain in clothing applications, we use repeated movements of stretching and relaxing the sensors. A Stanley Heavy Duty Utility Vice was used to stretch the sensors. To determine the maximum extension that the sample must be able to measure, we consider the extension of the lumbar spine (bent flexion) in cyclist jerseys, that is, 3 cm stretch over 20 cm of fabric. A fabric should hence reliably measure stretch up to 15%.

**Figure 5:**
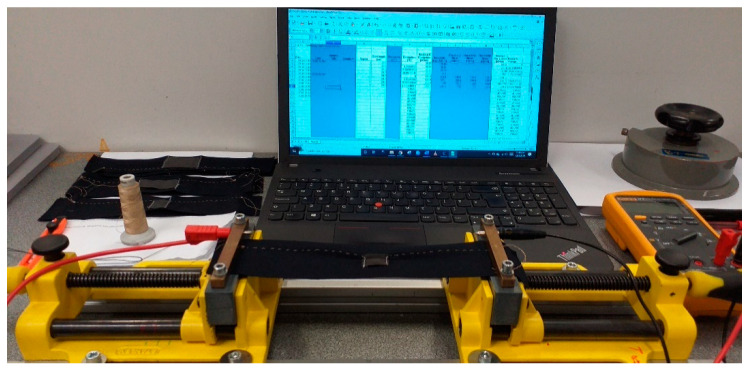
Experimental setup for the measurement of resistivity.

After the sample construction, the sensors’ working range, working function, resistivity, sensitivity and stability were determined. Each sample was digitally photographed, and the image saved with its code, e.g., I-1.jpg (Figure 4), in order to later be able to ascertain no visual changes occurred due to stretching. The sample is then placed on the vice 20.2 cm apart using the lines on the base fabric and given a small pretension. The test samples were next extended from 5% to 15% (1 cm to 3 cm) elongation, which is assumed a typical body movement for the 20.2 cm initial length, and the change in resistance is recorded (Figure 5). We waited for 30 s, 60 s and 90 s after reaching the extension, press hold on the multimeter, and record the resistance in a spreadsheet for later analysis.

### 2.4. Characterization of Test Specimens

- Resistivity at varying sample size

The total resistivity between the clamps at rest is recorded to obtain the difference due to the sample size. With this test, some insights can be obtained on the influence of the measurement range on the conductivity characteristics of the fabric.

- Resistivity at different elongation

This experiment should confirm the stress resistivity characteristic and show the influence of stress on the sensor while maximum or minimum tension is applied. The sensor was tested with 0%, 5%, 10% and 15% stretch of the base fabric to which the sensor is attached. The extension varied from 1 cm to 3 cm considered the typical lumbar ROM.

- Resistivity at varying waiting times (stability)

This test is done to see the stability of the resistivity of the sensor at every level of extension. The effect of waiting times on resistivity characteristics of the sensor was observed at 30, 60 and 90 s. From this, we obtain if there is an influence of time on the resistivity as well as sensitivity features when waiting for a longer period.

- Sensitivity

The sensitivity of sensor resistance to strain changes is defined as a measure of how much the resistance changes in percentage per elongation percentage during strain, and it is calculated using Equation (1). It indicates the overall sensing property of the sensor, where a high value indicates the sensor is very sensitive:

(1) $$\mathrm{Sensitivity}=\;\frac{\frac{R_{N}-R_{0}}{R_{0}}}{\frac{L_{N}-L_{0}}{L_{0}}},\;$$

where *R_N_* = the resistance at *N* cm elongation; *R*_0_ = resistance at 0 cm elongation; *L_N_* = length at elongation *N* cm; *L*_0_ = length at 0 cm elongation.

- Sweat influence

An artificial sweat solution (0.9% sodium chloride + 100 mL of water) was prepared. Sweat is applied to the sensor by placing 1 mL on the sensor using a micropipette, and then the weight of the sample is determined to obtain the weight increase while wetted. Beforehand, the samples were kept 24 h prior to the test in the conditioned lab environment for acclimatization. Thereafter, the tests were performed as described in the previous sections to obtain the resistance values when sweat was added. The samples were dried after each test and the following tests start again from the dry samples.

## 3. Results and Discussion

In this section, the sensor working range, the sensor sensitivity, and dependency on the amount of extension (%) and waiting time are presented. The phenomenon of whether resistance increases or decreases with extension has also been analyzed. Further, to show the functioning of the textile sensor on the garment, the influence of a body sweat rich environment was examined and compared. As mentioned in the previous sections, the experiment was conducted to measure the resistivity performance of a strain/stretch sensor which is developed from conductive textiles. With the analyzed data comparison was made between samples to determine the optimized sample type and this sample type is then selected for measuring other parameters and to identify the potential factors that influence the resistivity performance.

### 3.1. Resistivity under Strain

The resistivity at varying sample size was as follows. For the sample I sensor, we obtained minimum and maximum average resistivity of 29.5 ± 2.47 kΩ and 33.7 ± 1.31 kΩ, while 54.4 ± 1.68 kΩ and 62.2 ± 3.36 kΩ for sample II and 42.3 ± 3.14 kΩ and 47.3 ± 3.79 kΩ for sample III respectively.

**Figure 6:**
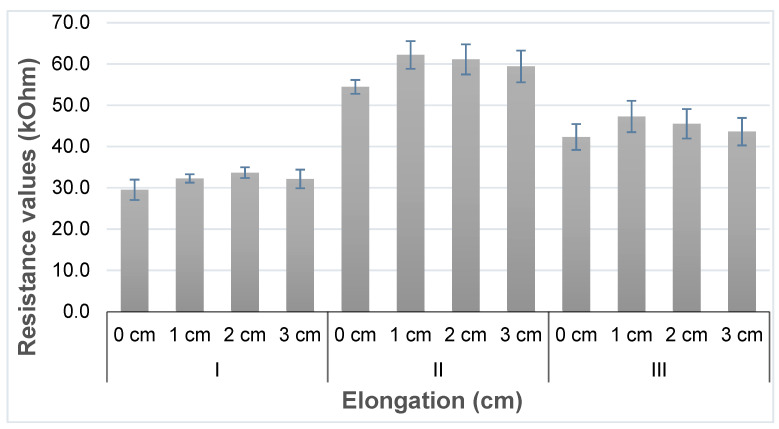
Average resistance response of the sensor to a given elongation and SEM.

**Figure 7:**
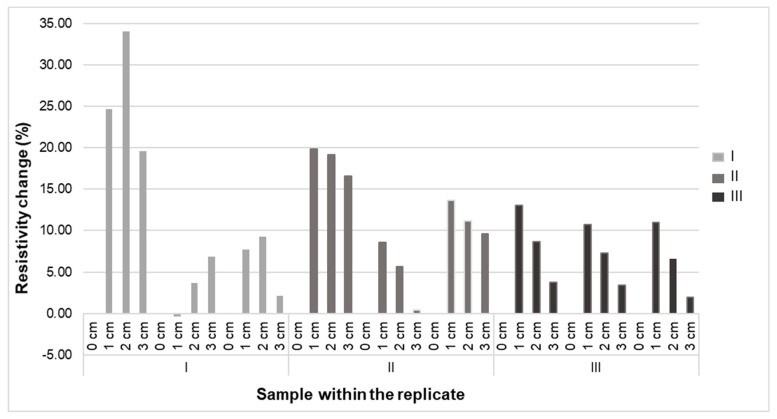
Sensitivity (%) the sensor to a sample under cyclic testing.

Next, for all sample types, the variation for a given elongation was measured (Figure 6). Error bars in the figure indicates the standard error of the mean (SEM) of the resistance values. The sensor was tested with 0%, 5%, 10% and 15% stretch, and underwent repeat cyclic testing. From the cyclic testing, a coefficient of variation (CV%) at 1 cm, 2 cm and 3 cm was calculated, and for sample type I, CV% (1.2, 1.03 & 0.95); for sample type II (0.4, 0.57 & 0.91) and for sample type III (0.11, 0.14 & 0.32) respectively was found. From this, we conclude that sample type III (sensor specimen length 5 cm) has the most stable operation, with least resistivity variation over the 3 replicates (Figure 7). Of the three types, it is hence the most suitable one for future use.

As explained in the methodology, tests where the elongation was gradually increased were conducted. The sensor first 0% (pre-stretched) to be stretched to its full work extension range before its first usage (pre-stretch). After that, the stretch was increased to 5%, 10% and 15%. As it can be seen from Figure 7, the maximum resistivity of the sensor was attained at 5% extension, after which the resistivity decreases up to 15%. This could be due to low initial resistance of short fibers, which will increase the change of resistance over initial resistance. These samples are knitted fabric, therefore, at first, the stretch will lead to a reorientation of yarns and loops. Apparently in a way that leads to higher resistivity. At higher stretch, however, the contact points start to press harder against each other, lowering the contact resistance, and hence decreasing the resistivity from that point onward.

This result means that this sensor should be used, or between 0 and 5% stretch, and over 5% stretch, as otherwise a resistance value cannot be linked to a unique strain. Therefore, with such characteristics, the EeonTex^TM^ (Eeonyx Corp., Pinole, CA 4564, USA) conductive stretchable fabric can only be used as a strain sensor with a minimum extension starting from rest, or as a sensor which is in a strained start state like in a compression garment, undergoing further stretch up to 15%.

Using sample type III, the following sensor properties are further examined: typical response of the sensor to a given elongation, dependency on the amount of extension (%), sensitivity, resistivity in pre-stretch and waiting time.

### 3.2. The Dependency of Resistivity over Waiting Time

**Figure 8:**
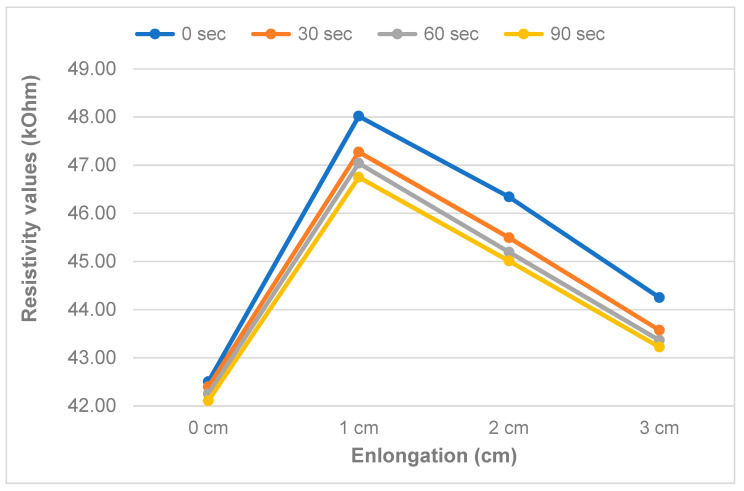
Response to a given extension with time (30 s interval) [type III].

In this section the phenomenon of whether resistance increases or decreases with strain/stretching has been analyzed. Sensor characteristics at different elongation percentage applied to the sensor are depicted. A typical resistance vs time plot is shown in Figure 8 in terms of the applied stretching for different waiting times at different extensions at 30 s intervals. It was found that the sensor needs to be stretched to its full work extension range before its first usage (pre-stretch). This allowed for the sensor properties to become stable. This means that during usage no larger strain than this pre-stretch value should be applied, and the pre-stretch should be chosen so as not to damage the material and allow for a full recovery after stretch.

**Figure 9:**
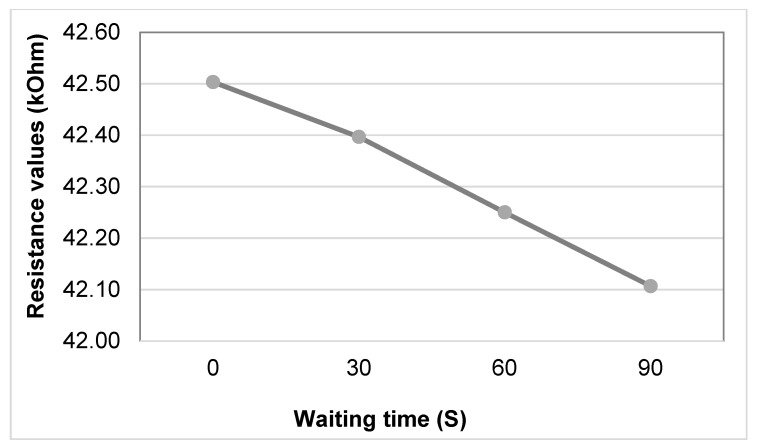
Resistance values of the sensor at 0% stretch after a pre-stretch of 15%.

The measured resistance varied between 42.11 kΩ at 0% stretch and 48.02 kΩ, where the maximum is reached at 5% stretch, and a decline is observed afterwards for the 10% & 15% stretch. Figure 8 illustrates that there is a rise in resistance when stretching up to 5% and a decrease afterwards. As it is clearly indicated in Figure 9, the output of the sensor drops with increasing waiting time, with the largest change during the first 30 s. This, however, means the sensor is not very suitable for dynamic environments, where we need to track fast body movements, and require sub-second readings of the sensor. The sensor requires slow-moving changes in stretch for accurate readings to be possible.

### 3.3. Dependency of the Sensor on Body Sweat

In order to show the usability of the sensor for clothing on the body which, especially during sports activity, is a sweat-rich environment, the sensor properties need to be stable when sweat is present in different concentrations. Therefore, a test was conducted where the sensor was exposed to sweat using artificial sweat (0.9% sodium chloride for 100 mL of water). On the sensor sample III (5 cm × 3 cm length), 1 mL of sweat was applied, and its weight in wet condition was measured. Then immediately following this sweat application testing was performed, so the sweat had no time to dry.

**Figure 10:**
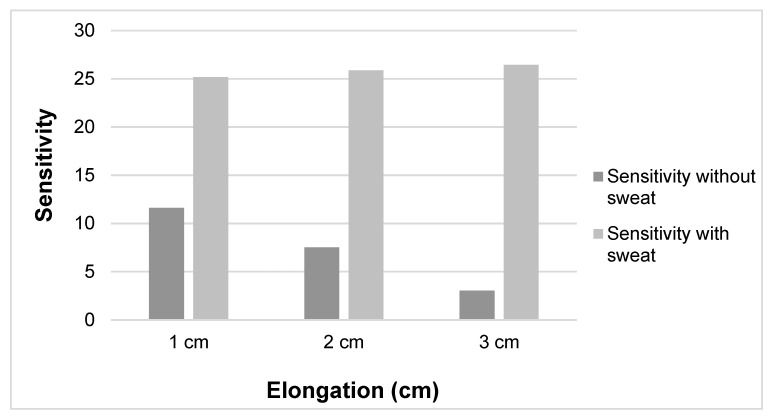
Sensitivity between sensor with and without sweat.

To determine the influence of body sweating on the resistivity characteristics, the tests with and without sweat were compared. As clearly seen in Figure 10, the sensitivity increased when the sensor had sweat present, but this sensitivity did not change much for the different extensions. The result shows there is only a slight rise in sensitivity when stretching up to 15% for a sensor with sweat and a clear decrease for a sensor without sweat. From the result, we can conclude that there are effects of strain on the sensor response.

**Figure 11:**
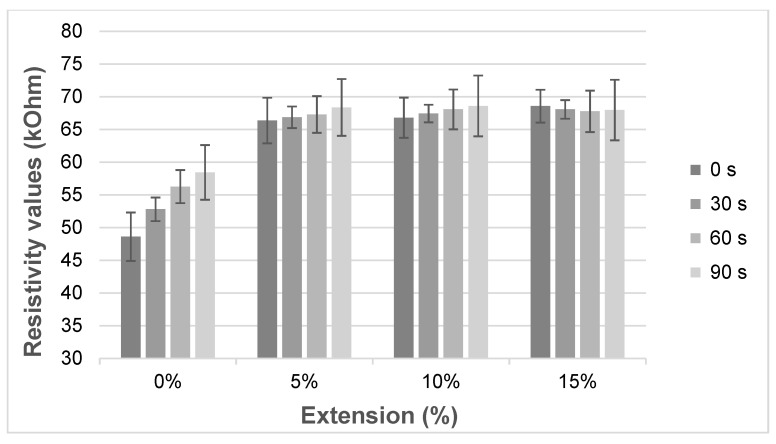
The resistivity for a given waiting time over elongation after sweat was applied.

The resistance values under extension at 30 s waiting time intervals are shown in Figure 11. They show that there is no big change in resistance value with the different extensions after 5% extension or more under all waiting times. The influence of elongation and waiting time on the resistivity were further analyzed to understand the dependency of resistivity on sweat.

**Figure 12:**
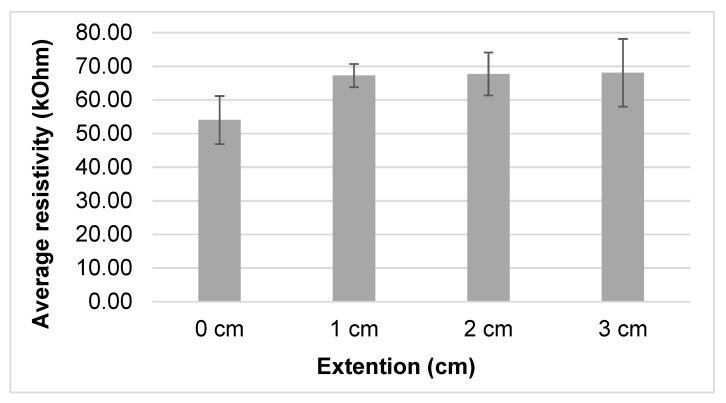
Average resistance value for a given elongation on repeat measurement at different waiting time.

Figure 11 illustrates the sensor response after sweat was applied with different waiting times. The minimal and maximal resistivity was 48.6 kΩ at 0 s with 0% stretch and 68.6 kΩ at 90 s with 10% stretching. Moreover, the average resistivity after different waiting times was calculated and found to be 54.03 kΩ with 0% stretch and 68.09 kΩ with 15% (Figure 12). The resistivity before pre-stretching was not stable with time (Figure 11, 0%), but this was not the case for the different elongations. The resistance increases over time for the 0, 5 and 10% case. The found variation over time is larger than the difference between the applied elongations, which means the sensor will not be able to distinguish different elongation states when sweat is present.

**Figure 13:**
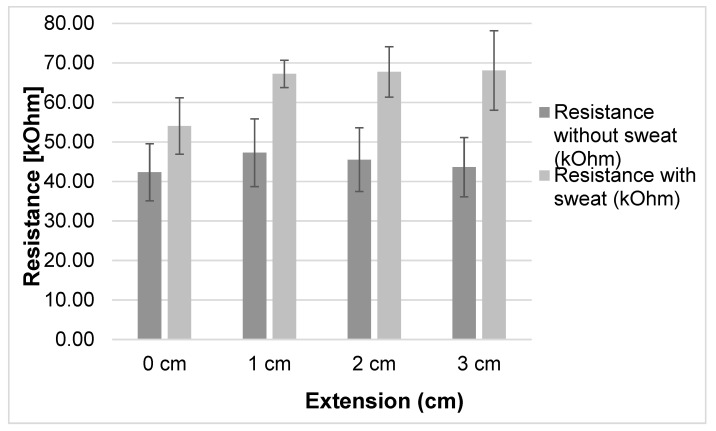
Resistivity response of sweated sensor vs non-sweated.

The results showed that resistance is higher when sweat is present and the sample is elongated (Figure 10). As clearly seen in Figure 10, the resistivity value of the conductive samples without sweat decreases as the elongation increases over 5%, as already shown also in Figure 8. This could be due to molecular rearrangement and a higher degree of orientation that improves the conductivity of material as the extra stretch is applied. For the samples which contain sweat, the resistance increases slightly with the extension (Figure 13), with average values 54.03, 67.23, 67.73 and 68.09 kΩ at 0, 5%, 10% and 15% stretch, respectively. This could be due to dimensional change, higher evaporation speed of sweat in nylon and molecular change due to the presence of sweat in the conductive yarn [32]. Another possible reason might be that EeonTex^TM^ conductive stretchable fabric is coated/doped with an inherently conductive polymer, making them conductive with quite high resistance. Such conductive polymers have varying resistance by varying the pressure, distance, thickness and stretchability [33,34]. Therefore, further study is needed to investigate the relation between sweat and conductive yarns/fabrics characteristics. These measurement variation ranges show the sensor should not be fully integrated into garment/textiles directly. Perhaps, additional processes are required (such as protective coating) to be able to obtain a sweat independent sensor which is protected from sweat penetration.

## 4. Conclusions

A stretchable conductive fabric changes resistance when stress/strain is applied, so it works as a strain sensor, but it also works as a force sensing resistor if the strain is the result of a force and Young’s modulus of the fabric is known. This study aimed to develop resistive strain sensors from stretchable conductive fabric and investigating the influence of stretchability (strain) over conductivity/resistivity. To see the potential of the sensor to use for measuring strains, different sensor properties were characterized. The sensor has a dynamic response to the elongation, which becomes mostly stable after 30 s, making the sensor suited for slow-moving changes, but not fast dynamic changes. Once stable, a rising resistivity is found up to 5% elongation, after which the resistivity drops again, indicating a usable region for the sensor of only up to 5%, or starting from 5% up to 15%, as otherwise, a resistivity value can indicate two different elongations, one below, and one over, 5%. It was further found that at fixed elongation, the sensor is not fully stable over time. Therefore, with such characteristics, the Eeon Tex^TM^ conductive stretchable fabric can only be used as a strain sensor with reduced accuracy. Commercially available strain gauges reach higher linearity but at a very reduced working range of less than 1% stretch. However, such small measurement ranges do not work for textile integrated applications. Of the three types tested, only the largest type, type III, was found to be suitable as a stretch sensor, however, this is less useful, as the larger area needed also means the resolution of the processes that can be tracked with the sensor is lower. It was also found that the textile stretch sensor has a slowly decaying resistance value overstretch, with the largest decrease during the first 30 s, making the sensor unsuitable for measuring fast-changing stretch conditions. Finally, for textile applications which are in contact with the human skin, the sensor should be sweat independent. Unfortunately, this was found not to be the case for this conductive fabric, allowing only applications which are not subjected to sweat. Overall, the fabric is not very suitable for most smart textile applications. The methods applied in this paper will be applied to other possible fabric stretch sensor to find a more suitable smart textile strain sensor. In literature, many conductive fabrics are presented with a resistive stretch effect. We advise that more thorough investigation is done on these by the creators, as in this paper, in order to determine if the stretch effect on resistivity is actually usable in a stretch sensor, or is an effect that can only determine some stretch is occurring, and nothing more.

## Figures and Tables

**Table 1:** Test number and sample size for the sensor fabric.

**Test No.**	**Sample Size for the Conductive Fabric**	
	**A (Wale)**	**B (Course)**
I	2 cm	2 cm
II	5 cm	2 cm
III	5 cm	3 cm
